# Early Screening of Sleep-Disordered Breathing Using a Smartphone-Based Portable System in Stroke Patients and Its Relevance for Rehabilitation: A Prospective Observational Study

**DOI:** 10.3390/s26030794

**Published:** 2026-01-24

**Authors:** Sergiu Albu, Yolanda Castillo-Escario, Alicia Romero Marquez, Mónica López Andurell, Raimon Jané, Hatice Kumru

**Affiliations:** 1 Institut Guttmann, Institut Universitari de Neurorehabilitació Adscrit a la UAB, 08916 Badalona, Spain; aromero@guttmann.com (A.R.M.); mlopez@guttmann.com (M.L.A.); hkumru@guttmann.com (H.K.); 2 Universitat Autónoma de Barcelona, 08193 Bellaterra, Spain; 3 Fundació Institut d’Investigació en Ciències de la Salut Germans Trias i Pujol, 08916 Badalona, Spain; 4 Department of Automatic Control, Universitat Politècnica de Catalunya-BarcelonaTech (UPC), 08028 Barcelona, Spain; ycastillo@ibecbarcelona.eu (Y.C.-E.); rjane@ibecbarcelona.eu (R.J.); 5 Institute for Bioengineering of Catalonia (IBEC), The Barcelona Institute of Science and Technology (BIST), 08028 Barcelona, Spain; 6 Centro de Investigación Biomédica en Red de Bioingeniería, Biomateriales y Nanomedicina (CIBER-BBN), 28029 Madrid, Spain

**Keywords:** stroke, sleep-disordered breathing, sleep apnea, nocturnal hypoxemia, portable device, functional outcomes

## Abstract

**Highlights:**

**What are the main findings?**

- The smartphone-based portable monitoring system enabled detection of previously undiagnosed sleep apnea among post-stroke patients undergoing rehabilitation.
- Greater sleep-disordered respiratory events and nocturnal hypoxemia were associated with worse baseline disability and lower rehabilitation metrics.

**What are the implications of the main findings?**

- The portable system was easy to use, facilitating sleep apnea detection after stroke and supporting broader implementation in rehabilitation settings.
- Routine screening for sleep-disordered breathing at admission may enable earlier diagnosis and management in patients with substantial hypoxemia/event burden that could slow functional recovery.

**Abstract:**

Sleep-disordered breathing (SDB) is common after stroke and may negatively influence recovery, yet it is frequently underdiagnosed. Portable respiratory monitoring devices could facilitate early SDB screening in these patients. We estimated the prevalence of sleep apnea (SA) using a smartphone-based monitoring system in post-stroke patients and examined associations between respiratory indices, stroke severity and disability (NIHSS, mRS), and rehabilitation outcomes (motor and cognitive Functional Independence Measure; FIM). Consecutive patients admitted to inpatient rehabilitation within three months after a stroke underwent an overnight assessment with a smartphone-based respiratory monitoring device, which estimated the apnea–hypopnea index (AHI), mean and minimum SpO_2_, time with SpO_2_ < 94% and <90%, and hourly oxygen desaturation events (≥3% and ≥4%). Of the 104 screened patients, 59 were recruited, while 56 had valid recordings. Most patients (89%) had previously undiagnosed SA: 11% mild (AHI ≥ 5 and <15), 38% moderate (AHI ≥ 15 and <30), and 41% severe (AHI ≥ 30). Greater event burden and nocturnal hypoxemia were associated with older age, worse baseline disability (mRS), lower admission motor FIMs, and poorer rehabilitation metrics. Smartphone-based portable monitoring is an accessible, easy-to-use approach that may enable earlier identification of SA, particularly in individuals with substantial hypoxemia or respiratory event burden.

## 1. Introduction

Sleep-disordered breathing (SDB) is a broad term that includes various conditions characterized by abnormal breathing patterns during sleep, such as obstructive sleep apnea (SA), central sleep apnea, and sleep-related hypoventilation [1]. A recent meta-analysis revealed a high prevalence of SA among stroke patients, with 71% of this population affected, of which 30% suffer from severe SA [2]. SA was more severe and over three times more prevalent in patients with brainstem involvement compared to those with other infarction sites [3].

SA often leads to excessive daytime sleepiness and fatigue, which can impair cognitive function and diminish a patient’s ability to participate fully in rehabilitation exercises, ultimately delaying their functional recovery [4,5,6]. Therefore, early diagnosis of SA and prompt therapeutic intervention may improve neurological outcomes and enhance the quality of life for stroke survivors.

Direct measurement of SDB is essential to accurately diagnose SA, since questionnaires have limited diagnostic accuracy for SA and may be challenging to complete for patients with stroke-related speech or cognitive impairments. According to the American Academy of Sleep Medicine (AASM), polysomnography (PSG) is the gold standard for diagnosing obstructive SA, but it is often impractical for widespread use because of the high prevalence of sleep disorders, the time-consuming nature of the test, and its associated costs. In cases where PSG is not feasible, such as during hospitalization, portable monitoring devices may be a reasonable alternative for assessing sleep-related respiratory disorders to facilitate timely therapeutic interventions [1].

Portable devices, such as home sleep apnea testing and pulse oximetry, have been shown to be feasible, cost-effective, and a sensitive screening tool for detecting SA in stroke patients and may serve as an effective preliminary method for selecting patients for more targeted diagnostic testing in the sleep laboratory and for therapeutic interventions for SA [7,8]. Moreover, smartphones have been proposed as low-cost, widely available tools to screen for SA by using built-in microphones, inertial sensors, and oximeters [9,10] and have been explored in preliminary studies to investigate SDB in patients with spinal cord injury [11] and stroke [12].

The primary objective of this study was to estimate the prevalence and severity of previously undiagnosed SDB in stroke patients admitted to rehabilitation using a portable smartphone-based monitoring device. Accordingly, the primary endpoint was the recording of the estimated apnea–hypopnea index (AHI) and its severity classification.

Secondary objectives were (1) to identify demographic, clinical, and stroke-related correlates of SA severity and nocturnal hypoxemia and (2) to explore associations between sleep-related respiratory metrics, functional status, and rehabilitation-related metrics during the same hospitalization period.

## 2. Materials and Methods

We conducted a prospective cohort study involving stroke patients who underwent inpatient rehabilitation at Institute Guttmann, a rehabilitation hospital in Barcelona (Spain) specializing in intensive rehabilitation of patients with neurological diseases.

The study was approved by the Research Ethics Committee of the Fundació La Unió, and all participants or their legal representatives provided written informed consent to participate. The study was carried out in accordance with the recommendations of the Declaration of Helsinki. The study conforms to the STROBE standards for observational studies (www.strobe-statement.org).

According to the prospective design, SDB was assessed once using a smartphone-based portable monitoring system early after admission, while functional and rehabilitation metrics were collected at admission and discharge as part of routine clinical care.

### 2.1. Participants

Eligible participants were adult patients over 18 years of age with ischemic or hemorrhagic stroke who were admitted for inpatient rehabilitation within three months of stroke onset between June 2023 and October 2024. Patients presenting with stroke secondary to traumatic brain injury, brain tumors, or infections were excluded from the study. In addition, we excluded patients previously diagnosed with SA, current Continuous Positive Airway Pressure (CPAP) users, patients with an active respiratory infection, those with post-stroke pulmonary thromboembolism, those carrying a tracheostomy tube or presenting with cardiac or respiratory conditions requiring invasive/non-invasive ventilation or oxygen support, and patients with a language barrier and no translation available.

### 2.2. Equipment and Instruments

The portable recording system consists of a Samsung Galaxy S5 SM-G900F smartphone running Android 6.0.1. (Samsung Electronics Co., Ltd. Suwon-si, Republic of Korea). The system allows for the simultaneous recording of three signals: audio, using the built-in high-quality microphone to capture environmental sounds, with a particular focus on snoring and respiratory sounds; a built-in triaxial accelerometer (MPU-6500 sensor, InvenSense, Inc. San Jose, CA, USA); and oxygen saturation (SpO_2_), using an external wireless EMO-80 fingertip pulse oximeter (EMAY Ltd., Hong Kong, China). The sampling frequency is 48 kHz for audio signals, 200 Hz for accelerometer data, and 1 Hz for SpO_2_. The data are automatically stored in the smartphone’s internal memory and can later be exported to a computer for reading and analysis.

The recording system was validated in previous studies, demonstrating accurate detection and severity stratification of all patients with sleep apnea (N = 13), with a concordance correlation coefficient of 0.99 for AHI estimation [9]. In addition to the agreement in the predicted AHI, an event-by-event analysis showed a sensitivity of 76% and a precision of 82%, and the system achieved a classification accuracy of 82% in classifying apneas and hypopneas [9]. In another validation study, the smartphone demonstrated 95.9% accuracy in determining body position compared to video-validated polysomnography [13]. Furthermore, this system was used to characterize SDB in hospitalized stroke patients [12]. However, it is important to note that our study proposes an early screening approach, similar to the role of polygraphy in clinical practice, and does not aim to replace PSG.

### 2.3. Evaluation of SDB

**Figure 1:**
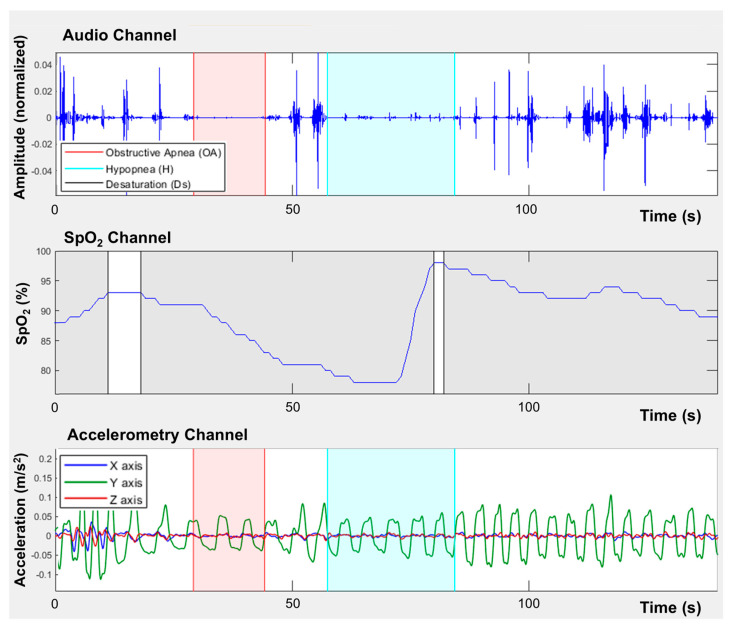
Example of smartphone-based recorded audio, SpO_2_, and accelerometer signals with identification of apnea and hypopnea periods.

The smartphone is placed on the chest and secured with an elastic band around the thorax at the sternum level. In this position, parallel to the frontal plane of the body, the built-in accelerometer detects chest movements during breathing as well as turns in bed. The microphone records snoring and breathing sounds, while the pulse oximeter records blood oxygen saturation. Data from the external pulse oximeter were sent to the smartphone via Bluetooth, and the device clock was synchronized with the phone at pairing to align timestamps. Figure 1 shows an example of the recorded audio, SpO_2_, and accelerometer signals.

### 2.4. Signal Processing and Analysis

Recordings were considered valid for evaluation only if they provided at least 4 h of usable overnight data.

The primary endpoint was to obtain an estimation of the apnea–hypopnea index (AHI) based on the recording time. For that purpose, the estimated AHI was calculated as the total number of automatically detected apneas and hypopneas divided by the total valid recording time (events/h). Valid recording time was defined after excluding the first 10 min of recording and removing all periods in which the patient was not lying in bed, as identified by accelerometer data. Because total sleep time and sleep stages were not measured, this index is not equivalent to the PSG-derived AHI and should be interpreted as a screening metric. Audio signals were downsampled to 5 kHz using anti-aliasing low-pass filtering and denoised using spectral subtraction [14] based on an automatically estimated noise model derived from low-energy segments in the initial recording period to suppress cardiac and ambient noise while preserving breathing and snoring components [12]. Apnea and hypopnea events were automatically detected via an entropy-based analysis of the smartphone acoustic signals, which identified respiratory events capturing characteristic reductions in sound intensity [9]. The apnea–hypopnea index (AHI) was defined as the total number of apneas plus hypopneas per hour; the apnea index (AI) is defined as the number of apneas per hour; and the hypopnea index (HI) is defined as the number of hypopneas per hour. Severity categories (no, mild, moderate, and severe SA) were applied using standard event-rate thresholds to facilitate clinical interpretability and comparisons with prior portable-monitoring studies while acknowledging the methodological differences in the PSG-based AHI. AHI severity was classified using standard cutoffs: No SA is AHI < 5 events/h. Mild SA is AHI ≥ 5 to <15 events/h. Moderate is AHI ≥ 15 to <30 events/h, and severe is AHI ≥ 30 events/h [15].

Secondary endpoints included oxygenation-derived metrics. The following oximetry-derived outcomes were computed: mean SpO_2_ as the primary measure of average oxygenation (AvgSpO_2_); minimum SpO_2_ as the lowest valid saturation level recorded (MinSpO_2_); cumulative time spent with SpO_2_ below 94% (CT94) and below 90% (CT90); and oxygen desaturation indices (ODI3 and ODI4), defined as the number of desaturation events with drops of SpO_2_ ≥ 3% and ≥4% per hour, respectively. These thresholds were selected to capture clinically relevant nocturnal hypoxemia (CT94) beyond the AHI [16], while the time spent below 90% additionally reflects cumulative hypoxic burden, which has been identified as an important risk factor for all-cause mortality [17].

Oral versus nasal breathing was determined from the acoustic spectrum by dividing the audio into 10 s windows, computing the FFT, and smoothing the spectrum using a linear envelope (15 Hz windows). A window was classified as oral breathing when the maximum spectral peak within 950–2000 Hz reached at least 60% of the maximum envelope value; otherwise, it was classified as nasal breathing. The resulting oral breathing metric was reported as the percentage of the recording time classified as oral breathing.

Body position during sleep was derived from accelerometer orientation with respect to gravity by computing a sleep angle in the X–Z plane and a stand angle in the Y–Z plane. Position-related metrics were used to quantify the proportion of time spent in the supine position (%) and the relationship between respiratory events and the supine position (AHI in the supine position).

### 2.5. Demographic and Stroke-Related Variables

We collected the following variables: demographic data [age and sex], stroke type [ischemic, hemorrhagic, or ischemic with hemorrhagic transformation], stroke location [supratentorial: hemispheric or brainstem], and laterality [right, left or bilateral].

Clinical scales were used to assess stroke severity on admission using the National Institutes of Health Stroke Scale (NIHSS) [18], and the degree of independence was measured using the Modified Rankin Scale (mRS) [19].

### 2.6. Cardiovascular Risk Factors and Medication

Cardiovascular risk factors (hypertension, atrial fibrillation, ischemic heart disease, diabetes mellitus, dyslipidemia, obesity, and currently smoking) were recorded as binary variables (“yes/no”). Obesity was defined as Body Mass Index (BMI) > 30 kg/m^2^, which was determined according to the WHO criteria.

### 2.7. Sleep Assessment Questionnaires

The sleep-related questionnaires were only administered to patients with preserved verbal or written comprehension abilities.

The Pittsburgh Sleep Quality Index (PSQI) is a self-administered questionnaire designed to evaluate sleep quality. It consists of 18 questions assessing seven-component scores (sleep quality, sleep latency, sleep duration, habitual sleep efficiency, sleep disturbances, use of sleeping medication, and daytime dysfunction). Each component’s score ranges from 0 to 3 (0, not in the past month; 1, less than once per week; 2, once or twice per week; and 3, three or more times/week). The 7-component scores are then summed to calculate a global PSQI score, which ranges from 0 to 21, with higher scores indicating poorer sleep quality [20].

The Patient-Reported Outcomes Measurement Information System (PROMIS) Sleep Disturbance Scale and the Sleep-related Impairment Scale integrate the qualitative aspects of sleep disturbance and sleep-related impairments across various clinical settings and a wide range of health conditions [21]. The short form of the PROMIS Sleep Disturbance Scale is an 8-item tool that evaluates self-reported perceptions of sleep quality, depth, and restoration associated with sleep over the past 7 days. Each item is rated on a 5-point Likert scale, from 1 to 5, with higher total scores indicating greater sleep disturbance [22]. Similarly, the short form of the Sleep-Related Impairment Scale is an 8-item measure that assesses alertness, sleepiness, tiredness, and functional impairments related to sleep problems during waking hours over the past 7 days. Participants rate each item on a scale from 0 (“not at all bothered”) to 5 (“very much bothered”), with higher scores indicating greater sleep impairment [22].

### 2.8. Rehabilitation-Related Indices

Rehabilitation variables included motor and cognitive Functional Independence Measure scores (m-FIM and c-FIM) at admission and discharge [23], as well as hospital length of stay (LOS). We derived indices of functional improvement: FIM gain was calculated as FIM at discharge − FIM at admission. m-FIM effectiveness (%) was calculated as m-FIM gain/(91 m FIM at admission). c-FIM effectiveness (%) was calculated as c-FIM gain/(35 − c-FIM at admission) to account for state-dependent recovery. FIM efficacy (%) was calculated as FIM gain/LOS to account for time-dependent recovery.

### 2.9. Statistics

Statistical analyses were conducted with a commercial Statistical Package for Social Sciences version 16.0.1 (SPSS Inc., Chicago, IL, USA, 2007).

Descriptive statistics were used for demographic and clinical characteristics. Continuous variables with an approximately normal distribution are presented as age-adjusted means with 95% confidence intervals, whereas categorical variables are presented as numbers and percentages. The Shapiro–Wilk test was used to examine the normality of the distribution. We used One-way ANCOVA to compare continuous variables between groups while adjusting for age as a covariate. Other clinically relevant variables (BMI, NIHSS, mRS, FIM, and cardiovascular comorbidities) were not included simultaneously to avoid model saturation, despite their known association with nocturnal hypoxemia. When variables did not meet parametric assumptions, we applied the most appropriate transformation to approximate normality (square-root, logarithmic, logit, or Box–Cox transformation) prior to analysis. Post hoc pairwise comparisons were performed with Bonferroni correction to adjust for multiple testing. The Chi-square test was applied to test relationships between categorical variables and SA severity groups.

Correlations between sleep-related breathing parameters, stroke severity, and functional outcomes were examined using Spearman’s rank correlation coefficient. These were considered exploratory. To mitigate multiple-testing concerns, we additionally applied FDR correction (Benjamini–Hochberg) across the correlation tests; FDR-adjusted q-values are reported for inference.

## 3. Results

A total of 104 patients were initially screened, of whom 10 were excluded as they were already using CPAP therapy due to a prior diagnosis of SDB before stroke. An additional 35 patients were excluded based on other exclusion criteria. Overnight recordings were conducted on 59 patients who were eligible for the study. However, the recordings failed in three patients, leading to their exclusion. Ultimately, 56 stroke patients (64% male, 36% female; mean age 55 ± 13 years) were included in the study, and FDR-adjusted *q*-values are reported for inference.

### 3.1. Prevalence and Severity of SDB Using Smartphone-Based Portable Systems

**Figure 2:**
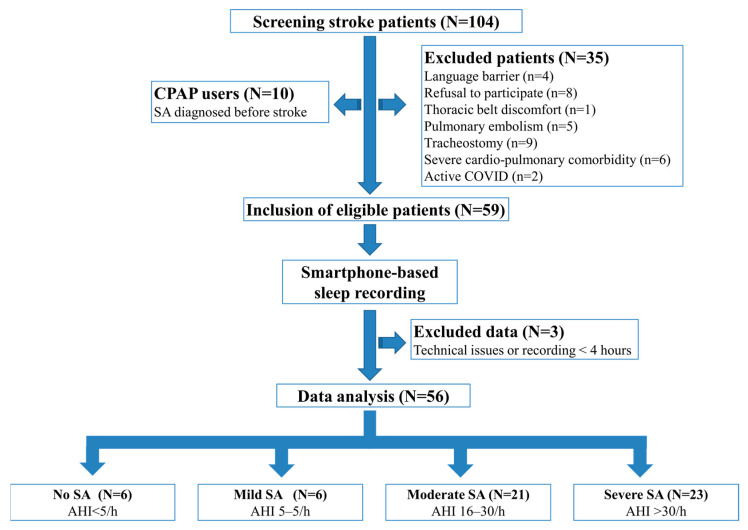
Flow diagram.

Based on the AHI, 6 patients (10.7%) had no SA, 6 (10.7%) had mild SA, 21 (37.5%) had moderate SA, and 23 (41.1%) had severe SA (Figure 2). Because the no-SA and Mild-SA groups were similar in terms of age, gender, stroke type, stroke severity (NIHSS), and functional status (FIM at admission), patients were classified into three main groups: Mild/no-SA (AHI < 15), Moderate-SA (AHI ≥ 15 < 30) and Severe-SA (AHI ≥ 30) (Table 1).

Patients’ ages were different among groups (F (2, 53) = 7.85, *p* = 0.001); therefore, age was included as a covariate in subsequent between-group ANCOVA analyses.

The AHI, AI, and HI increased progressively across sleep apnea severity groups (Table 2). As SA severity increased, average and minimum SpO_2_ levels tended to decline. Average SpO_2_ levels differed across SA groups (Mild/no-SA: 95 [94, 97], Moderate-SA: 93 [92, 94], Severe-SA: 93 [92, 94]; F(2, 52) = 3.69, *p* = 0.03). The Bonferroni post hoc test showed a significant difference only between severe SA and mild/no SA (*p* = 0.03). Minimum SpO_2_ levels also varied by severity (Mild/no-SA: 84 [78, 91], Moderate-SA: 82 [77, 86], Severe-SA: 74 [69, 78]; F(2, 52) = 4.56, *p* = 0.015), with the Bonferroni post hoc test again indicating a significant difference only between severe SA and mild/no SA (*p* = 0.02).

Both oxygen desaturation indices increased with SA severity: ODI3 (Mild/no-SA: 8 [0, 15], Moderate-SA: 22 [17, 27], Severe-SA: 45 [40, 50]; F(2, 52) = 40.99, *p* < 0.001) and ODI4 (Mild/no-SA: 4 [−3, 12], Moderate-SA: 13 [7, 18], Severe-SA: 34 [29, 39]; F(2, 52) = 33.09, *p* < 0.001). Bonferroni-adjusted post hoc tests indicated significant differences between all groups for both indices (all *p* < 0.001).

Greater nocturnal hypoxemia was evident in higher-severity groups: CT94 (Mild/no-SA: 26 [8, 44], Moderate-SA: 45 [33, 58], and Severe-SA: 48 [35, 61]; F(2, 52) = 3.89, *p* = 0.03) and CT90 (Mild/no-SA: 2 [−9, 12], Moderate-SA: 7 [0, 14], and Severe-SA: 17 [9, 24]; F(2, 52) = 7.36, *p* = 0.002). Bonferroni post hoc tests showed longer time < SpO_2_ 94% in Moderate-SA versus Mild/no-SA (*p* = 0.03) and longer time < SpO_2_ 90% in Severe-SA versus Mild/no-SA (*p* = 0.001) and Moderate-SA versus Mild/no-SA (*p* = 0.04); other pairwise comparisons were not significant after adjustment.

The AHI in the supine position was higher in more severe groups (Mild/no-SA: 10 [2, 17], Moderate-SA: 22 [17, 27], and Severe-SA: 48 [43, 53]; F [2, 52) = 32.47, *p* < 0.001), with Bonferroni-adjusted post hoc tests showing significant differences for all pairwise comparisons (all *p* < 0.001). In contrast, there was no evidence of a severity effect on oral breathing (*p* = 0.94) or on time spent in the supine position (*p* = 0.19).

### 3.2. Sleep Questionnaires

Based on reports from patients or their family members, previously suspected sleep-apnea tended to be more common with greater SA severity (Mild/no-SA: 8%; Moderate-SA: 43%; Severe-SA: 48%)(χ^2^(2) = 5.66, *p* = 0.06) (Table 3).

Use of sleep medication was more frequent in the less severe groups (Mild/no-SA: 83%, Moderate-SA: 71%, and Severe-SA: 61%) and was not statistically significant (χ^2^(2) = 1.93, *p* = 0.38). The most frequently used sleep medications were trazodone (59%), mirtazapine (21%), clonazepam (15%), quetiapine (10%), lorazepam (10%), lormetazepam (8%), melatonin (5%), and clorazepate (3%), gabapentin (3%), and diazepam (3%).

Sleep questionnaires were not available in 29% of patients with stroke-related speech or cognitive impairments. After controlling for age, the absolute time spent in bed (hours) did not differ across SA groups (Mild/no-SA: 9 [8, 10] h, Moderate-SA: 9 [9, 10] h, and Severe-SA: 9 [9, 10] h; F(2, 36) = 0.61, *p* = 0.55). Self-reported sleep duration was likewise non-significant (Mild/no-SA: 6 [5, 7] h, Moderate-SA: 8 [7, 8] h, and Severe-SA: 7 [7, 8] h; F(2, 36) = 2.20, *p* = 0.13). Sleep efficiency (sleep duration/ time in bed) also did not differ across groups (Mild/no-SA: 72 [60, 84]%, Moderate-SA: 83 [74, 92]%, and Severe-SA: 80 [70, 89]%; F(2, 36) = 0.96, *p* = 0.39).

Consistent with this pattern, sleep-quality questionnaires showed poorer perceived sleep in the Mild/no-SA group than in the Moderate-SA and Severe-SA groups; however, omnibus tests for PSQI global scores, PROMIS impairments, and disturbance scores were not significant (all *p* > 0.05) (Table 3).

### 3.3. Clinical and Stroke-Related Characteristics Across SA Severity Groups

The proportion of male patients was higher in the Moderate-SA (76%) and Severe-SA (65%) groups compared to the Mild/no-SA group (42%), although this difference was not statistically significant (χ^2^(2) = 3.98, *p* = 0.14). Ischemic stroke was the most prevalent type in the Moderate-SA (57%) and Severe-SA (57%) groups, while hemorrhagic stroke was more common in the Mild/no-SA group (58%). Ischemic stroke with hemorrhagic transformation occurred exclusively in the Moderate-SA (5%) and Severe-SA (17%) groups (χ^2^(4) = 5.92, *p* = 0.21). Most lesions were unilateral hemispheric (Mild/no-SA (75%) and Moderate-SA (76%) and Severe-SA (83%)), while brainstem strokes were less frequent (17%, 24%, and 13%, respectively) (χ^2^(6) = 5.03, *p* = 0.54).

Exploratory analysis at admission revealed that stroke severity, as measured by the NIHSS, was similar across groups: (Mild/no-SA: 13 [9, 18], Moderate-SA: 11 [8, 14], and Severe-SA: 13 [10, 15]; F(2, 42) = 0.34, *p* = 0.72). Likewise, baseline disability was comparable between groups: most patients presented moderate-to-severe disability (mRS 4–5), with no significant differences in mRS distribution across groups (χ^2^(4) = 6.57, *p* = 0.16). Functional Independence Measure (FIM) scores at admission were also similar across SA severity groups: m-FIM (Mild/no-SA: 50 [36, 63], Moderate-SA: 46 [37, 55], and Severe-SA: 39 [30, 49]; F(2, 52) = 0.86, *p* = 0.43) and c-FIM (Mild/no-SA: 26 [21, 31], Moderate-SA: 23 [19, 26], and Severe-SA: 25 [21, 29]; F(2, 52) = 0.75, *p* = 0.48).

The prevalence of cardiovascular risk factors was generally higher in the Moderate-SA and Severe-SA groups. However, only dyslipidemia (χ^2^(2) = 10.84, *p* = 0.04) and arrhythmia (χ^2^(2) = 6.18, *p* = 0.045) showed a statistically significant association with more severe SA (Table 1).

Motor and cognitive FIM at discharge, as well as motor and cognitive FIM gain, effectiveness, and efficacy) did not significantly differ across SA groups (all *p* > 0.05). The length of hospital stay was similar in all groups (*p* = 0.62) (Table 4).

### 3.4. Exploratory Correlation Analysis Between Smartphone-Based Respiratory Metrics, Stroke Severity, and Rehabilitation Metrics

As shown in Table 5, older age was associated with more severe respiratory disturbance and hypoxemia. Higher BMI showed the same pattern, as it was associated with worse respiratory indices.

Lower motor FIM on admission was associated with greater oral breathing (ρ = −0.35, *p* = 0.008, q = 0.03), while c-FIM at admission showed no significant associations with any respiratory variables (Table 5).

c-FIM gain was reduced with lower night-time oxygenation: AvgSpO_2_ (ρ = 0.40, *p* = 0.002, q = 0.01) and CT94 (ρ = −0.38, *p* = 0.004, q = 0.015). Lower m-FIM effectiveness was associated with lower nocturnal oxygenation: CT90 (ρ = −0.37, *p* = 0.005, q = 0.02) and oral breathing (ρ = −0.37, *p* = 0.005, q = 0.02). c-FIM efficacy decreased with lower nocturnal oxygenation: CT94 (ρ = −0.39, *p* = 0.003, q = 0.013) and AvgSpO_2_ (ρ = 0.40, *p* = 0.002, q = 0.01).

No significant associations were found between respiratory indices and stroke severity (NIHSS) or sleep questionnaires.

## 4. Discussion

In this prospective observational study, the primary finding was a high prevalence of previously undiagnosed SA (89%) among post-stroke patients undergoing inpatient rehabilitation, as identified by a smartphone-based portable monitoring system, with moderate and severe SA identified in 79% of cases. SA severity, respiratory events, and hypoxemia were related to worse baseline disability and functional motor impairments at admission, as well as to lower functional recovery metrics, which highlights the need for early sleep disorder screening at admission. This underscores the importance of using portable and accessible devices as an effective screening method for SA in stroke patients, thereby facilitating early management and targeted interventions.

### 4.1. Sleep Apnea Prevalence and Severity in Stroke Patients

The global prevalence of obstructive sleep apnea (AHI > 5/h) is estimated at nearly 1 billion for people aged 30–69 years [24]. However, among stroke patients, the prevalence of SA is significantly higher. Our findings align with previous research on stroke patients. A recent meta-analysis reported that 71% of stroke patients experience SA (AHI > 5/h), with 30% presenting with severe SA (AHI > 30/h) [2]. Notably, SA remained consistently high from the acute (<1 month) to the subacute (1–3 months) and chronic phases (>3 months) after stroke, with the prevalence of mild, moderate, and severe SA ranging from 65 to 67%, from 33 to 50%, and from 25 to 36%, respectively [25].

The American Academy of Sleep Medicine recommends polysomnography (PSG) as the gold-standard diagnostic test for SA [1]. However, this method is expensive, technically complex, often unavailable in acute settings, and typically requires an overnight stay in a specialized sleep lab, which limits its accessibility for many patients. Portable devices are alternative methods to diagnose obstructive SA in adults. Respiratory polygraphy devices have been shown to effectively screen for SA in stroke patients, with no significant differences compared to PSG [2]. The European Academy of Neurology, the European Respiratory Society, the European Sleep Research Society, and the European Stroke Organization advocate the use of portable cardiorespiratory polygraphy as a practical alternative for assessing the prevalence and severity of SA in post-stroke patients [26]. Our study demonstrates the practical applicability of a portable sleep monitoring device in stroke patients, supporting these recommendations and further highlighting their potential clinical utility for SA screening.

### 4.2. Role of Portable Systems in Sleep-Disordered Breathing Screening

Interestingly, self- or family-suspected SDB corresponded with a higher prevalence, as confirmed by our portable smartphone-based monitoring system. In our study, self- or family-reported SDB was common in the Moderate-SA (42.9%) and Severe-SA (47.8%) groups. However, subjective reports can still underestimate the true disease burden: in a recent stroke cohort, only 23.3% of patients and 11.7% of caregivers independently reported sleep disturbances, yet PSG identified obstructive SA in 62% of those denying sleep problems and in 100% of those self-reporting these issues [27].

In the present work, the portable device was used as a screening tool to identify patients at increased risk of SDB rather than as a diagnostic surrogate for PSG. Our results highlight the value of portable sleep monitoring systems not only for identifying and classifying SA severity but also for quantifying hypoxemia. While the AHI/AI/HI clearly increased across severity groups, oxygenation metrics showed clinically meaningful worsening with severity (lower AvgSpO_2_ and minSpO_2_, higher ODI3 and ODI4, and longer CT94 and CT90). Incorporating these hypoxemia-related measures can better capture the physiological impact of SA and may support more informed early decisions about initiating CPAP in stroke patients.

The portable device used in our study also records body position, allowing us to examine how posture influences breathing during sleep. A large study in a non-stroke adult population found that side sleeping was the most common position (54.1%), followed by supine sleeping (37.5%); the proportion of time spent in the side position increased with both age and BMI [28]. On the contrary, after a stroke, patients may spend more time in the supine position, which can compromise upper airway patency and impair chest movements on the paralyzed side and respiratory function [29]. Our results show that patients spent on average between 56 and 74% of their sleeping time in the supine position. Furthermore, the supine AHI was higher in older patients, those with higher BMI scores, and those with more severe SA. This positional information can help identify patients at risk for obstructive SA and support targeted preventive measures (e.g., positional strategies) during inpatient rehabilitation [30].

### 4.3. Questionnaires and Smartphone-Based Sleep Measures

In our study, questionnaires were used to characterize self-perception of sleep quality, duration, and disturbance (PSQI) as well as sleep-related disturbances and impairments (PROMIS). However, questionnaires were not available in 29% of patients with stroke-related speech or cognitive impairment. Patients with more severe SA had lower PSQI and PROMIS sleep-related impairment and disturbance scores (although not statistically significant) and higher AHI and AI scores, which were correlated with lower PROMIS impairment scores. In a study evaluating sleep quality perception among obstructive SA patients, more severe hypoxemia was associated with better perceived sleep quality, probably due to hypoxemia-related impairment of perception [31]. However, PSQI is not helpful in the pre-polysomnographic assessment of people with suspected obstructive SA [32]. There is a strong recommendation against using questionnaires in the absence of PSG or portable devices to diagnose obstructive SA in adults.

### 4.4. Role of Stroke Characteristics and Associated Risk Factors on Sleep Apnea

Age (≥55 years old), male sex, or post-menopausal status in women are relevant well-known risk factors for obstructive SA [33]. Our findings are consistent with previous reports: patients with severe SA were significantly older than those with mild/no SA, and male sex was more predominant in the Moderate- and Severe-SA groups. Furthermore, objective measures obtained with the smartphone-based portable system indicated that older age and higher BMI scores were associated with more severe respiratory disturbance and greater hypoxemia.

Obstructive SA is recognized as an independent risk factor for stroke [34]. However, the relationship between obstructive SA and stroke may also be bidirectional, since both conditions share common risk factors, including age, hypertension, dyslipidemia, diabetes mellitus, atrial fibrillation, obesity, and unhealthy lifestyle habits such as a poor diet, smoking, and alcohol consumption [35]. In our study, hypertension, dyslipidemia, arrhythmia, obesity, and diabetes mellitus were more prevalent in patients with severe SA. These findings highlight the importance of addressing the management of modifiable risk factors, which could potentially significantly improve outcomes for both SA and stroke patients.

Several studies have explored the relationship between stroke severity, stroke location, and the prevalence or severity of sleep apnea. In a large acute-stroke cohort, NIHSS scores at admission did not differ between patients with and without obstructive SA [36], and a meta-analysis likewise found no differences by stroke severity; prevalence was numerically higher in supratentorial vs. infratentorial strokes, but it was not statistically significant [37]. Consistent with this, we found no significant differences in stroke type, stroke severity (NIHSS), or location across SA groups. Notably, ischemic stroke with hemorrhagic transformation only occurred in the Moderate- and Severe-SA groups, a complication linked to a higher risk of adverse events, longer hospitalization, death, and worse outcomes at discharge [38]. In our study, worse disability (higher mRS and lower m-FIM) at admission was associated with greater nocturnal hypoxemia.

Sleep medication was used by 70% of patients in our study. Although sedative–hypnotic drugs have historically been avoided in obstructive SA because of concerns that suppression of arousal and upper-airway muscle activity could worsen hypoxemia, current evidence suggests that although hypnotics may increase the respiratory arousal threshold, they do not consistently modify obstructive SA severity, with meta-analytic data showing no significant overall change in AHI with hypnotic use [39]. In our cohort, the most frequently used sleep medications were trazodone (59%) and mirtazapine (21%), whereas benzodiazepines were used by fewer than 15% of patients. This medication profile is relevant because trazodone is among the few agents with the potential to improve the AHI, respiratory arousal, and nocturnal hypoxia in stroke patients [40], whereas benzodiazepines show no significant overall change in AHI and a near-zero net effect on genioglossus responsiveness, refuting earlier concerns about clinically relevant muscle relaxation [39]. Therefore, despite the high prevalence of hypnotic/psychotropic use, sleep medication use in our study is unlikely to have substantially biased SA severity estimates, reinforcing the robustness of our sleep-disordered breathing evaluation in this population.

### 4.5. Sleep-Disordered Breathing and Rehabilitation-Related Metrics

As reported, between-group comparisons showed no statistically significant differences in rehabilitation outcomes across sleep apnea severity groups. The exploratory correlational analyses described several associations, but they are insufficient to imply causality or exert an influence on recovery. Admission mRS was correlated with multiple oxygenation metrics (ODI4, Avg SpO_2_ CT94 and CT90), suggesting that a higher hypoxemia score is present in more dependent patients at admission to a rehabilitation hospital. Importantly, lower nocturnal oxygenation and higher respiratory event burden were also associated with less favorable rehabilitation change metrics (c-FIM gain, m-FIM effectiveness, and c-FIM efficacy).

These findings align with prior evidence indicating that SDB is highly prevalent in both the acute phase and at 3 months after a stroke/transient ischemic attack, consistent with a potential contribution of pre-existing SDB to stroke and post-stroke disability; the AHI has also been proposed as a predictor of more severe disability (mRS ≥ 3), reinforcing the value of SDB screening in stroke patients [41].

In prior work focusing on sleep duration, sleep quality, and sleep-related impairment—rather than sleep apnea severity measured by the AHI—broader sleep problems have been associated with worse outcomes in stroke survivors, including impaired balance and gait [42], poorer recovery across domains such as activities of daily living/instrumental activities of daily living, mobility, cognition, communication, and social participation [5], and worse neurological recovery at 3 months in those with poor sleep quality or an abnormal sleep duration [43]. In patients with SA, a meta-analysis reported that positive airway pressure therapy reduced recurrent vascular events and improved neurological deficit, cognition, and functional independence [44].

Although functional metrics were assessed at discharge, this study was not designed to establish causal effects of SDB on rehabilitation outcomes. The exploratory correlation analyses focused on associations between respiratory metrics and functional status and on admission and recovery indices observed during the same inpatient rehabilitation period. Therefore, these correlations should be interpreted as indicative of clinically relevant relationships rather than direct outcome effects.

### 4.6. Study Relevance

Our study contributes to existing knowledge by demonstrating the high prevalence of undiagnosed and untreated SA in hospitalized stroke patients and the practical use of smartphone-based portable technology for SA screening in a rehabilitation setting.

Taken together, our results highlight the need to improve screening protocols and to better integrate sleep assessments into routine stroke rehabilitation, thereby enabling timely identification of SA, referral for PSG, and specialist evaluation when indicated.

One of the unique strengths of our study is its focus on the 1- to 3-month post-stroke period, which is particularly critical for stroke recovery [45]. Providing a screening tool to identify stroke patients with moderate to severe SA early during admission to rehabilitation may enable prompt clinical management.

### 4.7. Study Limitations

There are several limitations to this study. While the prospective design of this study provides methodological strengths, including the use of a standardized evaluation protocol for SDB, high-quality recorded data, and reduced selection bias, the relatively small sample size, restricted to hospitalized patients from a single center, may limit the generalizability of the findings to the broader population of post-stroke patients.

Furthermore, in our study older age predicted more severe sleep apnea. However, older adults (>60 years) were underrepresented in the current study. According to the Cerebrovascular Diseases Master Plans of the Health Department of Catalonia, elderly stroke patients who are unable to participate in an intensive rehabilitation program, due to associated medical conditions and premorbid functional status, are mainly candidates for rehabilitation in an extended care facility [46]. Therefore, the prevalence of SA in older post-stroke patients could be underestimated.

Although clinically relevant variables such as BMI, NIHSS, mRS, FIM, and cardiovascular comorbidities are known to be associated with hypoxemia and respiratory burden, the relatively small and unbalanced sample sizes across sleep-disordered breathing groups necessitated a conservative modeling approach. Accordingly, the number of covariates included in the models was limited to reduce the risk of overfitting and unstable parameter estimates.

An important methodological limitation is that sleep stages and total sleep time were not measured, and respiratory events were derived from acoustic and oximetry signals over the total sleep recording time. While this system is a practical and useful tool for assessing SA, it is not PSG, the gold-standard test for sleep apnea, which should be considered when interpreting our findings. Nonetheless, we used a smartphone-based portable system that was previously employed in stroke patients [12] and was validated in patients with SA using a standard polygraphy device [9,13]. The smartphone-based system should therefore be regarded as a screening tool rather than a diagnostic substitute for PSG. These factors should be considered when interpreting the screening results. Furthermore, relying on self-reported sleep data in patients with even mild cognitive impairment may also have introduced some bias regarding the sleep quality evaluation. These challenges further support the use of this technology as a screening tool to identify patients who may benefit from a formal sleep evaluation.

## 5. Conclusions

Undiagnosed sleep apnea is highly prevalent among post-stroke patients undergoing in-hospital rehabilitation. Smartphone-based portable monitoring appears to be a feasible screening approach for identifying sleep-disordered breathing in this clinical setting. Exploratory analyses suggest associations of apnea severity and hypoxemia with markers of stroke severity and rehabilitation-related metrics. Further adequately powered studies using comprehensive sleep assessments are needed to clarify the clinical impact of sleep-disordered breathing on rehabilitation outcomes in this population.

## Figures and Tables

**Table 1:** Sociodemographic and clinical characteristics of stroke patients.

Variables	Mild/no-SA (N = 12)	Moderate-SA (N = 21)	Severe-SA (N = 23)	*p* Value, One-Way ANCOVA	*p* Value, Chi Squared Test
**Age**	43 [34, 51] ^a^	53 [47, 59]	60 [55, 65]	0.001	
**Sex: Male (%)**	5 (42%)	16 (76%)	15 (65%)		0.14
**Stroke Type**					
Ischemic	5 (42%)	12 (57%)	13 (57%)		0.21
Hemorrhagic	7 (58%)	8 (38%)	6 (26%)		
Ischemic with hemorrhagic transformation	0 (0%)	1 (5%)	4 (17%)		
**Stroke to admission time (days)**	38 [25, 51]	48 [39, 57]	53 [44, 62]	0.21	
**Stroke Location**					
Right hemisphere	4 (33%)	10 (48%)	7 (30%)		0.54
Left hemisphere	5 (42%)	6 (29%)	10 (43%)		
Bilateral hemispheres	1 (8%)	0 (0%)	3 (13%)		
Brainstem	2 (17%)	5 (24%)	3 (13%)		
**NIHSS at admission**	13 [9, 18]	11 [8, 14]	13 [10, 15]	0.72	
**mRS at admission**					
mRS = 3 (%)	3 (25%)	2 (10%)	4 (17%)		0.16
mRS = 4 (%)	7 (58%)	14 (67%)	8 (35%)		
mRS = 5 (%)	2 (17%)	5 (24%)	11 (48%)		
**m-FIM at admission**	50 [36, 63]	46 [37, 55]	39 [30, 49]	0.43	
**c-FIM at admission**	26 [21, 31]	23 [19, 26]	25 [21, 29]	0.48	
**Smoker status**					
Ex-smoker	1 (8%)	2 (10%)	6 (26%)		0.17
Never Smoker	7 (58%)	6 (29%)	9 (39%)		
Active Smoker	4 (33%)	13 (62%)	8 (35%)		
**Hypertension**	5 (42%)	12 (57%)	17 (74%)		0.16
**Arrhythmia**	0 (0%)	0 (0%)	4 (17%) ^b^		0.045
**Diabetes Mellitus**	1 (8%)	4 (19%)	8 (35%)		0.18
**Dyslipidemia**	0 (0%)	5 (24%)	12 (52%) ^b^		0.004
**Ischemic heart disease**	1 (8%)	2 (10%)	1 (4%)		0.79
**Obesity**	2 (17%)	6 (29%)	10 (43%)		0.25
**BMI**	25 [22, 28]	27 [25, 29]	27 [25, 29]	0.44	

Continuous variables are reported as adjusted means with 95% confidence intervals derived from the age-adjusted ANCOVA model, whereas categorical variables are reported as counts (n) and percentages (%). One-way ANCOVA was used for between-group comparisons of continuous variables. The Bonferroni-corrected comparisons showed the following: ^a^ *p* < 0.001: Mild/no-SA vs. Severe-SA. ^b^ *p* < 0.05: The Chi-squared test indicates significant relationships between categorical variables.

**Table 2:** Smartphone-based respiratory metrics.

Variables	Mild/no-SA (N = 12)	Moderate-SA (N = 21)	Severe-SA (N = 23)	*p* Value, One-Way ANCOVA
**AHI (events/h)**	6 [0, 12] ^ab^	22 [18, 26] ^c^	50 [46, 54]	<0.001
**AI (events/h)**	2 [−3, 7] ^ab^	9 [6, 13] ^c^	19 [16, 22]	<0.001
**HI (events/h)**	6 [1, 12] ^ab^	13 [9, 17] ^c^	27 [24, 31]	<0.001
**ODI3**	8 [0, 15] ^ab^	22 [17, 27] ^c^	45 [40, 50]	<0.001
**ODI4**	4 [−3, 12] ^ab^	13 [7, 18] ^c^	34 [29, 39]	<0.001
**CT94**	26 [8, 44] ^d^	45 [33, 58]	48 [35, 61]	0.03
**CT90**	2 [−9, 12] ^ef^	7 [0, 14]	17 [9, 24]	0.002
**Average SpO_2_**	95 [94, 97] ^g^	93 [92, 94]	93 [92, 94]	0.03
**Minimum SpO_2_**	84 [78, 91] ^h^	82 [77, 86]	74 [69, 78]	0.015
**Oral Breathing (%)**	27 [15, 38]	30 [22, 38]	31 [22, 39]	0.94
**Time in supine position (%)**	65 [47, 83]	56 [43, 69]	77 [64, 89]	0.19
**AHI in supine position (%)**	10 [2, 17] ^ab^	22 [17, 27] ^c^	48 [43, 53]	<0.001

Variables are reported as adjusted means with 95% confidence intervals derived from the age-adjusted ANCOVA model. For variables bounded at 0, 95% CIs are model-based and may be negative due to sampling uncertainty and the linear-model formulation; this does not indicate negative observed values. One-way ANCOVA was used for between-group comparisons of continuous variables. The Bonferroni-corrected comparisons showed the following: ^a^ *p* < 0.001: Mild/no-SA vs. Moderate-SA; ^b^ *p* < 0.001: Mild/no-SA vs. Severe-SA; ^c^ *p* < 0.001: Moderate-SA vs. Severe-SA; ^d^ *p* = 0.03: Mild/no-SA vs. Moderate-SA; ^e^ *p* = 0.04: Mild/no-SA vs. Moderate-SA; ^f^ *p* = 0.001: Mild/no-SA vs. Severe-SA; ^g^ *p* = 0.03: Mild/no-SA vs. Severe-SA; and ^h^ *p* = 0.02: Mild/no-SA vs. Severe-SA.

**Table 3:** Questionnaire-based sleep metrics.

Variables	Mild-No SA (N = 12)	Moderate-SA (N = 21)	Severe-SA (N = 23)	*p* Value, One-Way ANCOVA	*p* Value, Chi Squared Test
**Sleep medication (%)**	10 (83%)	15 (71%)	14 (61%)		0.38
**Suspected sleep apneas (%)**	1 (8%)	9 (43%)	11 (48%)		0.06
**Time spent in bed (hours) ^¥^**	9 [8, 10]	9 [9, 10]	9 [9, 10]	0.55	
**Self-reported sleep time (hours) ^¥^**	6 [5, 7]	8 [7, 8]	7 [7, 8]	0.13	
**Sleep efficiency (%) ^¥^**	72 [60, 84]	83 [74, 92]	80 [70, 89]	0.39	
**PROMIS sleep impairment ^¥^**	18 [14, 22]	14 [11, 17]	13 [10, 16]	0.21	
**PROMIS sleep disturbance ^¥^**	19 [14, 23]	19 [15, 22]	17 [13, 20]	0.67	
**Pittsburgh total score ^¥^**	11 [8, 14]	7 [5, 9]	8 [5, 10]	0.20	

Continuous variables are reported as adjusted means, with 95% confidence intervals derived from the age-adjusted ANCOVA model, whereas categorical variables are reported as counts (n) and percentages (%). One-way ANCOVA was used for between-group comparisons of continuous variables, along with Bonferroni-adjusted post hoc tests. The Chi-squared test was used to study the relationships between categorical variables. ^¥^ Data were available for 71% of patients: Mild–no-SA (N= 10), Moderate-SA (N = 15), and Severe-SA (N = 15).

**Table 4:** Rehabilitation-related metrics.

Variables	Mild/no-SA (N = 12)	Moderate-SA (N = 21)	Severe-SA (N = 23)	*p* Value, One-Way ANCOVA
**m-FIM at admission**	50 [36, 63]	46 [37, 55]	39 [30, 49]	0.43
**c-FIM at admission**	26 [21, 31]	23 [19, 26]	25 [21, 29]	0.48
**m-FIM at discharge**	75 [60, 89]	63 [53, 73]	61 [51, 71]	0.26
**c-FIM at discharge**	30 [25, 34]	26 [23, 30]	28 [25, 32]	0.50
**m-FIM gain**	25 [16, 34]	17 [11, 24]	21 [15, 28]	0.18
**c-FIM gain**	4 [1, 6]	4 [2, 6]	3 [1, 5]	0.77
**m-FIM effectiveness**	65 [46, 83]	53 [40, 65]	45 [32, 57]	0.16
**c-FIM effectiveness**	48 [25, 71]	35 [19, 50]	54 [38, 70]	0.11
**m-FIM efficacy**	40 [24, 56]	29 [18, 40]	30 [18, 41]	0.33
**c-FIM efficacy**	5 [−1, 10]	6 [3, 10]	6 [3, 10]	0.48
**LOS**	72 [55, 90]	65 [53, 77]	70 [58, 83]	0.62

Variables are reported as adjusted means, with 95% confidence intervals derived from the age-adjusted ANCOVA model. For variables bounded at 0, 95% CIs are model-based and may be negative due to sampling uncertainty and the linear-model formulation; this does not indicate negative observed values. One-way ANCOVA was used for between-group comparisons of continuous variables, along with Bonferroni-adjusted post hoc tests.

**Table 5:** Exploratory correlation analysis between smartphone-based respiratory metrics, socio-demographic, stroke severity, sleep questionnaires, and rehabilitation-related metrics.

	**Age**	**BMI**	**NIHSS**	**mRS**	**m-FIM Admission**	**c-FIM Admission**	**m-FIM Gain**	**c-FIM Gain**	**m-FIM Effectiveness**	**c-FIM Effectiveness**	**m-FIM Efficacy**	**c-FIM Efficacy**	**PROMIS Impairment**	**PROMIS Disturbance**	**PSQ Total Score**	0.6	Spearman rho (only significant FDR shown)
**AHI**	0.42 **	0.30	−0.04	0.18	−0.17	0.18	0.06	−0.10	−0.20	0.12	0.08	−0.12	−0.33	−0.29	−0.19	0.5	
**AI**	0.38 *	0.33 *	−0.08	0.27	−0.25	0.07	−0.02	−0.07	−0.27	0.00	−0.01	−0.06	−0.32	−0.27	−0.19	0.4	
**HI**	0.52 ***	0.36 *	0.04	0.16	−0.17	0.13	0.12	−0.04	−0.09	0.06	0.04	−0.07	−0.16	−0.18	−0.13	0.3	
**ODI3**	0.49 ***	0.41 **	0.07	0.32	−0.30	0.08	−0.01	−0.05	−0.31	0.05	−0.07	−0.09	−0.28	−0.25	−0.17	0.2	
**ODI4**	0.52 ***	0.44 **	0.05	0.33 *	−0.28	0.07	−0.02	−0.06	−0.31	0.02	−0.10	−0.10	−0.30	−0.25	−0.18	0.1	
**CT94**	0.47 ***	0.48 ***	0.10	0.37 *	−0.26	0.09	−0.07	−0.38 *	−0.28	−0.17	−0.12	−0.39 *	−0.23	−0.07	−0.09	0	
**CT90**	0.52 ***	0.49 ***	0.11	0.40 **	−0.28	0.11	−0.09	−0.22	−0.37 *	−0.04	−0.13	−0.23	−0.18	−0.20	−0.04	−0.1	
**AvgSpO_2_**	−0.46 **	−0.51 ***	−0.12	−0.40 **	0.25	−0.15	0.10	0.40 **	0.31	0.14	0.15	0.40 **	0.23	0.13	0.14	−0.2	
**MinSpO_2_**	−0.45 **	−0.41 **	−0.04	−0.27	0.25	−0.16	0.00	0.06	0.27	−0.11	0.13	0.14	0.08	0.26	0.10	−0.3	
**Oral Breathing**	0.24	0.03	0.19	0.25	−0.35 *	−0.04	−0.12	−0.10	−0.37 *	0.00	−0.16	−0.12	−0.02	−0.03	0.12	−0.4	
**Supine time (%)**	−0.11	−0.05	0.15	0.04	−0.11	0.09	0.22	0.10	−0.04	0.19	0.17	0.01	−0.08	−0.30	−0.06	−0.5	
**AHI in supine**	0.50 ***	0.28	−0.01	0.23	−0.18	0.11	0.01	−0.05	−0.16	0.02	−0.01	−0.04	−0.22	−0.20	−0.19	−0.6	

Values are Spearman’s rank correlation coefficients (ρ). Statistical significance is assessed using FDR-adjusted q-values (Benjamini–Hochberg); unadjusted *p*-values are not used for inference. Only correlations that remain significant after FDR correction (q < 0.05) are marked in the heat map. * q < 0.05, ** q < 0.01, and *** q < 0.001.

## Data Availability

The research data supporting the results reported in this article are available upon request from the Research Department of the Institute Guttmann (investigacio@guttmann.com).

## Abbreviations

The following abbreviations are used in this manuscript:

AHI	Apnea–Hypopnea Index
AI	Apnea Index
BMI	Body Mass Index
c-FIM	Cognitive Functional Independence Measure
CPAP	Continuous Positive Airway Pressure
CT90	Cumulative time with SpO_2_ < 90%
CT94	Cumulative time with SpO_2_ < 94%
FIM	Functional Independence Measure
HI	Hypopnea Index
LOS	Length of hospital stay
m-FIM	Motor Functional Independence Measure
MinSpO_2_	Minimum oxygen saturation
mRS	Modified Rankin Scale
NIHSS	National Institutes of Health Stroke Scale
ODI3	Oxygen Desaturation Index (drops of SpO_2_ ≥ 3% per hour)
ODI4	Oxygen Desaturation Index (drops of SpO_2_ ≥ 4% per hour)
PSG	Polysomnography
PROMIS	Patient-Reported Outcomes Measurement Information System
PSQI	Pittsburgh Sleep Quality Index
SA	Sleep apnea
SDB	Sleep-disordered breathing
SPSS	Statistical Package for the Social Sciences
SpO_2_	Peripheral capillary oxygen saturation
STROBE	Strengthening the Reporting of Observational Studies in Epidemiology
